# Interfacial Adhesion between Fatty Acid Collectors and Hydrophilic Surfaces: Implications for Low-Rank Coal Flotation

**DOI:** 10.3390/molecules27144392

**Published:** 2022-07-08

**Authors:** Yangchao Xia, Dan Fang, Pengcheng Qu, Yonggai Li

**Affiliations:** 1 National Engineering Research Center of Coal Preparation and Purification, China University of Mining and Technology, Xuzhou 221116, China; fdan1106@163.com (D.F.); ts21040091a31tm@163.com (P.Q.); 2 School of Chemical Engineering and Technology, China University of Mining and Technology, Xuzhou 221116, China; liyonggai@cumt.edu.cn

**Keywords:** flotation, low-rank coal, fatty acid collector, computer simulation, force measurement

## Abstract

Fatty acids, which are enriched in vegetable oil, have attracted much attention in low-rank coal flotation because of their unique chemical structure. In this study, density functional theory calculations, molecular dynamics simulations, and atomic force microscopy were employed to investigate the adsorption structure and forces between collectors and hydrophilic surfaces. The results show that fatty acids can be easily adsorbed onto surfaces through hydrogen bonds, and can cover the oxygen sites. The existence of hydration film on hydrophilic surfaces prevented nonpolar molecules from being able to adsorb, while polar fatty acids could adsorb and expel water molecules. The adhesion force between the RCOOH-terminated probe and the surface appeared in the retraction process, which differed significantly from that of the RCH_3_-terminated probe, indicating that polar fatty acids are more suitable as flotation collectors for low-rank coal than nonpolar hydrocarbon oil. The simulation and AFM test revealed the mechanisms of polar fatty acids, and can provide guidance for low-rank coal flotation applications.

## 1. Introduction

Low-rank coal reserves are abundant in China, the United States, Russia, and Australia. Low-rank coal includes lignite, long-flame coal, non-sticky coal, weakly sticky coal, and other low metamorphic coals, accounting for approximately half of all known coal reserves [1,2]. With the improvement of coal mining mechanization, the deterioration of coal-seam geological conditions, and the wide application of dense medium cyclones, the proportion of fine particles of low-rank coal has increased sharply, and ash content has also shown a deteriorating trend [3]. Owing to the characteristics of high water and ash content, the use of low-rank coal is limited [4]. Arbitrary stacking occurs in many underdeveloped places, which not only wastes resources, but also causes soil, river, and air pollution [5,6]. Flotation is among the most economical and effective methods for separating impurities in fine coal [7,8,9,10]. However, there are a large number of polar chemical groups on the surface of low-rank coal, resulting in hydrophilic sites [11,12,13,14,15]. Effective adsorption of the hydrocarbon oil onto hydrophilic sites of coal, along with significant improvement of the floatability of the coal surface, remains challenging [16,17,18].

At present, many scholars believe that adding surfactants or polar collectors can significantly improve the hydrophobicity and floatability of low-rank coal [19,20,21,22]. Chander et al. [23] improved the surface hydrophobicity of coal particles by adding a block copolymer. Block copolymers can be selectively adsorbed at hydrophobic and hydrophilic sites. Polat et al. [24] believed that the addition of surfactants could effectively reduce the amount of conventional oil collectors. Jia et al. [25] studied the effect of a series of tetrahydrofurfuryl esters on the flotation of low-rank coal, and concluded that tetrahydrofurfuryl ester collectors could bond with oxygen-containing functional groups and benzene rings on the surface of low-rank coal, with stronger hydrogen and π bonding. Cebeci [26] found that when emulsifiers and combinations of emulsifiers and surfactants were added as collectors, the recovery of combustible matter was significantly higher than that of kerosene alone. Chang et al. [27] pointed out that the proportion of TX-100 should increase with an increase in the oxidation of coal during flotation. It can be seen that the adsorption of polar agents on the oxygen-containing sites on the coal surface can effectively enhance the flotation recovery, but the adsorption mechanism of collectors on the oxygen-containing sites is still lacking.

The development of computational chemistry and the development of advanced testing equipment have enabled the study of micro-interface phenomena [28,29,30,31]. Zhang et al. [32] used density functional theory (DFT) to reveal the mechanism of action between oxidized coal and one nonpolar molecule and five polar molecules. Xia et al. [22] used molecular dynamics (MD) simulations to find that oily reagents can easily self-aggregate into spheres in a water environment, and clarified the reason as to why conventional oil collectors cannot effectively spread on the coal surface. Xing et al. [33] tested the interaction between polar and nonpolar reagents and hydrophilic oxidized coal with the help of colloidal-probe atomic force microscopy (AFM), and found that there was always a repulsive effect between the nonpolar reagent and the oxidized coal, while there was a strong adhesion effect between the polar reagent and the coal. Wu et al. [34] studied the adhesion between mica surfaces and different functional groups using chemical force microscopy. The results showed that the adhesion between the mica and the functional groups decreased in the following order: -NH_2_ > -COOH > -CH_3_ > -C_6_H_5_. Feng et al. [35] used dodecane as a model oil, and used droplet colloid probe technology to test the interaction force between nonpolar oil and a molybdenite base surface. The results showed that the force curve was in good agreement with the Reynolds lubrication model, amplified the Young-Laplace equation, and dominated the adhesion process. Molecular simulation technology and AFM tests have been successfully used to study flotation interfaces.

Fatty acids, which are enriched in vegetable oil, have been used in the study of low-rank coal flotation in recent years. In view of the lack of a fatty acid strengthening mechanism for the flotation of low-rank coal, the chemical adsorption structure and force properties between polar collector molecules and oxygenated carbonaceous surfaces were studied using computer simulation technology and AFM tests in this study.

## 2. Experimental Materials and Methods

### 2.1. Density Functional Theory (DFT) Calculation

**Figure 1:**
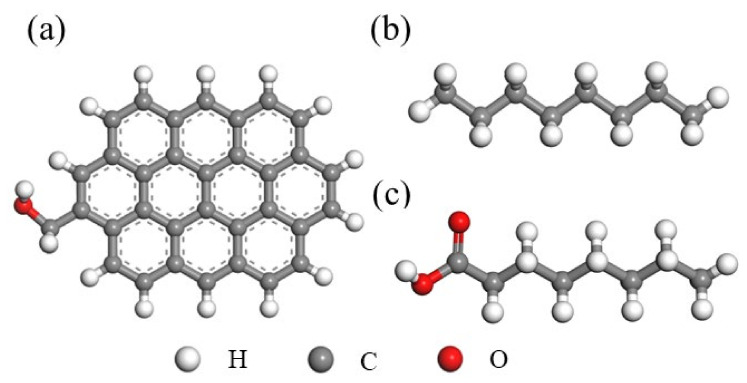
Molecular structure: (**a**) low-rank coal model; (**b**) nonpolar collector; (**c**) fatty acid collector.

The adsorption structure and energy of the nonpolar collector (octane) and the polar collector (octanoic acid) with the low-rank coal surface were calculated using DFT. In view of the complexity of the coal’s macromolecular structure and the operational ability of the computer, the aromatic ring bonded with the side chain and -OH was used as the hydrophilic low-rank coal surface, as shown in Figure 1. First, the low-rank coal and collector molecules were geometrically optimized, following which the optimal energy adsorption sites of collector molecules on low-rank coal were determined using the Monte Carlo method. After the adsorption site was determined, the low-rank coal-collector system was geometrically optimized to obtain the final adsorption structure and adsorption energy. Geometric optimization was conducted using the B3LYP functional method, double numerical polarization basis set, and unrestricted electron spin. The energy, maximum force, and maximum displacement were 2.0 × 10^−5^ ha, 0.004 ha/Å, and 0.004 Å, respectively. The DFT-D correction was used to describe weak interactions, such as hydrogen bonds. The solvation model was adopted, and the dielectric constant of the water was 78.54.

### 2.2. Molecular Dynamics (MD) Simulation

**Figure 2:**
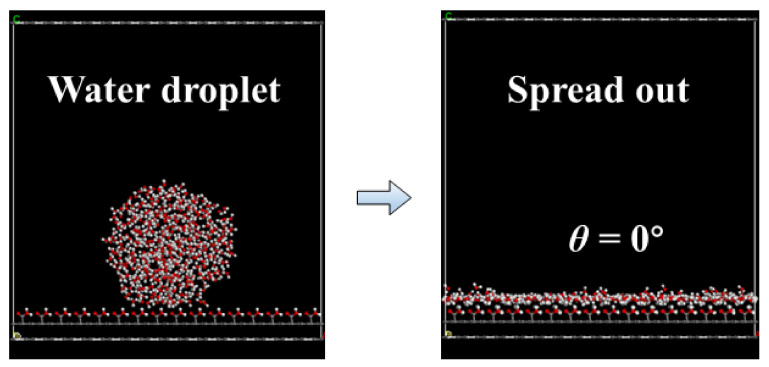
Hydrophilic -COOH surface used as an oxygenated carbonaceous surface, and the spreading process of water molecules.

The interfacial adsorption behavior of nonpolar and polar fatty acid collector molecules on the low-rank coal surface was studied using MD simulations. Owing to the complexity of the coal’s molecular structure, a flat, hydrophilic, oxygenated carbonaceous surface was constructed by COOH modification on a three-layer graphite surface. The collectors were undecane molecules and undecanoic acid molecules. The smart method was adopted to geometrically optimize the structure of the oxygenated carbonaceous surface, water molecules, undecane, and undecanoic acid. After geometric optimization, the oxygenated carbonaceous surface remained fixed. Then, the established water droplets with a radius of 15 Å were placed on the oxygenated carbonaceous surface to detect the surface contact angle. After geometric optimization, a molecular dynamics simulation of 500 ps was performed. It was observed that water droplets can completely spread on the -COOH-modified surface, as shown in Figure 2, which shows that the surface established in the simulation can replace the hydrophilic low-rank coal surface.

When calculating their adsorption structure, the optimized collectors were placed onto the oxygenated carbonaceous surface, and then water molecules were filled into the box through the packing function to establish the initial configuration, and the geometry of the system was similarly optimized. Subsequently, MD calculations of the adsorption of undecane and undecanoic acid molecules on the oxygenated carbonaceous surface in an aqueous environment were conducted.

The simulations were performed using the COMPASS force field. An NVT ensemble and Nosé temperature control method were selected. Above the simulation system, a vacuum layer of 50 Å was added along the z-direction. The interaction of electrostatic and van der Waals forces was calculated using the Ewald summation method, with a calculation accuracy of 0.0001 kcal/mol. The total simulation time was 1 ns, and the system reached equilibrium after 500 ps.

### 2.3. Atomic Force Microscopy (AFM) Test

**Figure 3:**
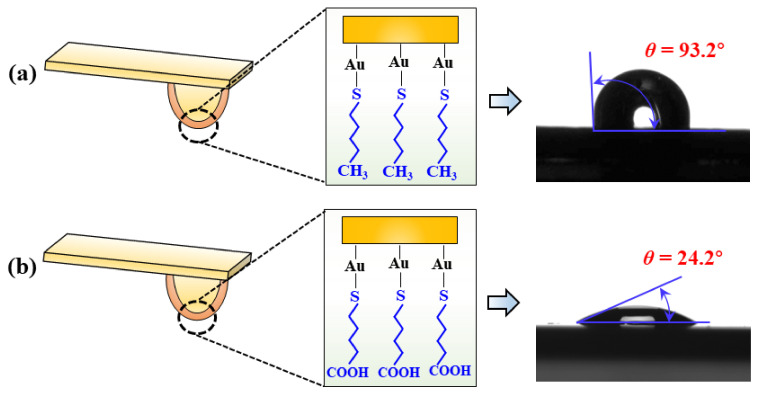
Schematic diagram of the (**a**) RCH_3_-terminated probe and (**b**) RCOOH-terminated probe, and their contact angles.

AFM was used to test the force curve between the collector molecules and the hydrophilic surface. Owing to the strict requirements of solid surface roughness for the AFM test, a flat SiO_2_ surface was used instead of a hydrophilic surface. Note that the collector molecules must be modified on the surface of the gold-plated probe tips before testing. The gold-plated tips can form stable Au-S covalent bonds with mercaptan groups; thus, mercaptan molecules with -CH_3_ and -COOH functional groups can be chemically modified to the probe surface. The gold-plated probe is shown in Figure 3. A PNP-TR-Au-20 probe with a nominal elasticity of 0.32 n/m was used. After cleaning the probe, it was immersed in 1-undecanethiol and 11-mercapto-undecanoic acid in ethanol solution at a concentration of 1 mM for 24 h to obtain the stable RCH_3_ and RCOOH functional group terminal probe tips, respectively. It should be noted that the same collectors as was used for flotation was not used in the preparation of the AFM-modified probe, because the modified probe, when prepared in that way, was unstable. The interaction force between RCH_3_ and the RCOOH terminal probe and surfaces was tested using a Bruker Multimode 8 AFM in the liquid-phase contact mode. During the test, the deflection sensitivity was measured first, and then the spot offset voltage signal detected by the optical detector was transformed into the actual bending amount of the cantilever. After updating the deflection sensitivity, the thermal tune procedure was used to calculate the cantilever’s elastic coefficient K. During the test, five force curves were collected at each position. Five different positions were collected for each surface by setting an offset of 500 nm to test the force curve to ensure the representativeness and accuracy of the force curve test results. A preparation diagram of the modified probe is shown in Figure 3.

### 2.4. Coal Surface Characteristics Tests

X-ray photoelectron spectroscopy (XPS, ESCALAB 250Xi, Thermo Scientific, Waltham, MA, USA) was used to analyze the surface chemical groups and elemental contents of the low-rank coal before and after the adsorption of octane and octanoic acid (2 kg per ton of coal). Al Kα radiation from a monochromatic X-ray source was used, and a light spot with a size of 900 μm was adopted during the test. Through a pass energy of 20 eV with a step of 0.05 eV, the high-resolution spectra were obtained.

### 2.5. Flotation Tests

**Figure 4:**
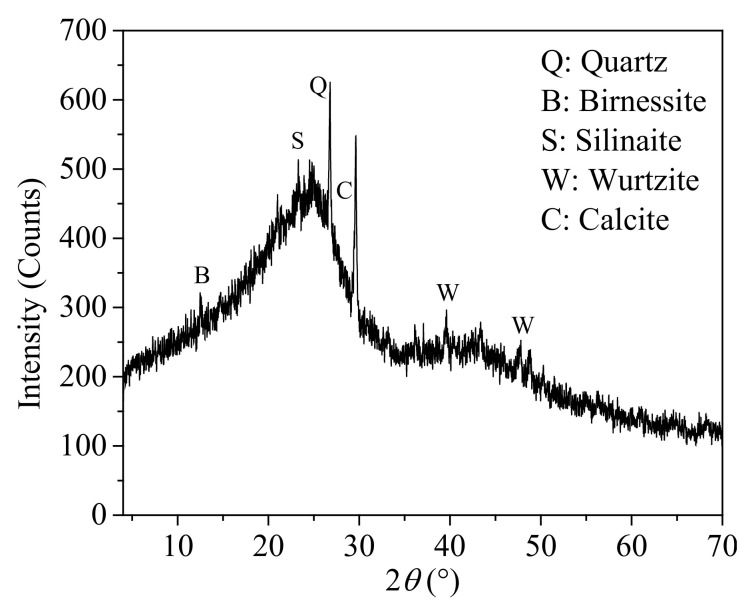
X-ray diffraction pattern of the low-rank coal.

A flotation experiment was carried out to observe the differences between nonpolar and fatty acid collectors. The particle size distribution of the coal samples and the composition of minerals in the coal are shown in Table 1 and Figure 4, respectively. The volume of the flotation cell was 1 L, and the coal concentration was 80 g/L. The flotation collectors were liquid octane (purity > 96%) and octanoic acid (purity > 98%). The amount of collector was set to 0.5, 1.0, 2, and 4.0 kg/t (that is, the amount of collector for one ton of dried coal). The dosage of frother remained fixed in all experiments (0.5 kg/t). All of the reagents were obtained from Aladdin Reagent Co., Ltd. (Shanghai, China) The impeller speed of the flotation machine was set to 1800 rpm. First, the fine coal was added to the cell for pulp conditioning for 120 s. Subsequently, the collector and frother were introduced for 120 s and 30 s, respectively. The airflow rate was set to 1 L/min, and then the concentrate and tailing products were collected over 300 s. The concentrate and tailing products were filtered and dried, and the concentrate was burned in a muffle furnace to determine the ash content.

## 3. Results and Discussion

### 3.1. Interaction between the Low-Rank Coal Surface and Collectors Using DFT Calculations

**Figure 5:**
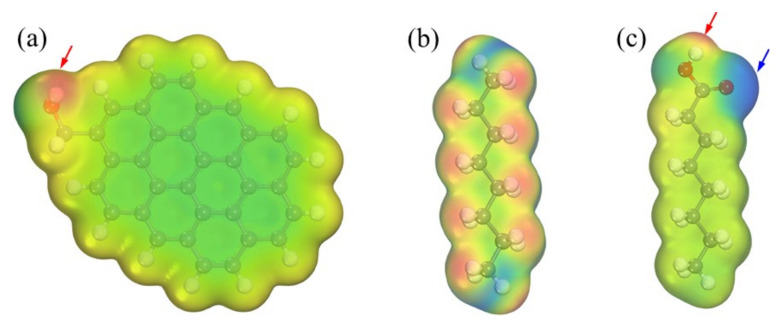
Distribution of electrostatic potential: (**a**) low-rank coal surface; (**b**) nonpolar collector; (**c**) fatty acid collector.

**Figure 6:**
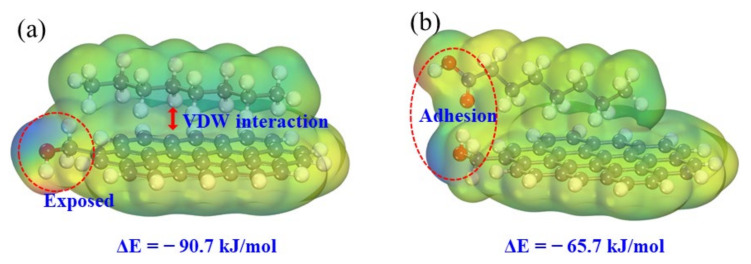
Electrostatic potential distribution of adsorption equilibrium structure: (**a**) nonpolar collector; (**b**) fatty acid collector.

DFT was used to calculate the electrostatic potential of the low-rank coal surface, nonpolar collector molecules, and fatty acid collector molecules, as shown in Figure 5. The electrostatic potential in the red area is positive. For example, near hydrogen atoms, the darker the color, the greater the electrostatic potential value, and the easier it is to obtain electrons. The electrostatic potential in the blue area is negative. For example, near the oxygen atom, the darker the color, the smaller the electrostatic potential, and the easier it is to lose electrons. It was found that the low-rank coal surface has positive and negative electrostatic potential extreme points; the nonpolar molecules have relatively uniform electrostatic potential distribution, and the fatty acid molecules also have positive and negative electrostatic potential extreme points. The equilibrium structure of the interaction between the collector molecules and the low-rank coal surface is shown in Figure 6. Because the nonpolar collector has no polar head group, it can only be adsorbed on the aromatic ring structure of oxygenated carbonaceous surface through the van der Waals force of the hydrophobic hydrocarbon chain, and the hydrophilic groups on the low-rank coal surface are still exposed. The fatty acid collector molecule has positive/negative electrostatic potential extreme points on the hydrophilic head group, which can be adsorbed on the negative electrostatic potential extreme points of the low-rank coal surface through hydrogen bonds. Although the interaction energy (∆E = −90.7 kJ/mol) between nonpolar collector molecules and the low-rank coal surface is greater than that between fatty acid collector molecules and the low-rank coal surface (∆E = −65.7 kJ/mol), nonpolar collector molecules cannot cover the oxygen-containing groups on the low-rank coal surface, and the hydrophobicity of the low-rank coal surface is still poor, while polar collector molecules can cover the oxygen-containing groups of the low-rank coal surface to achieve hydrophobic effects.

### 3.2. Molecular Adsorption Structure of the Collectors

**Figure 7:**
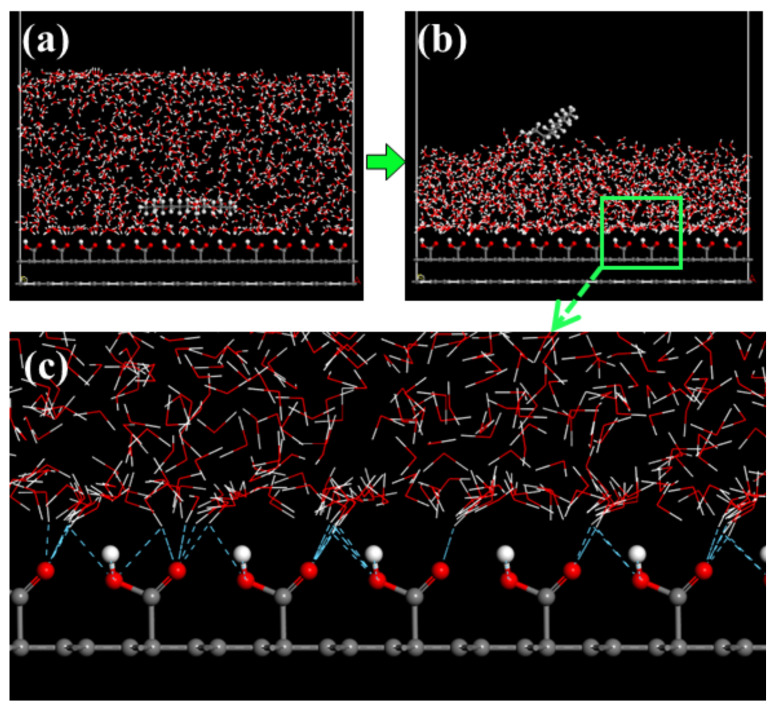
Adsorption of nonpolar collector molecules on a hydrophilic oxygenated carbonaceous surface: (**a**) initial structure; (**b**) balanced structure; (**c**) local enlargement.

**Figure 8:**
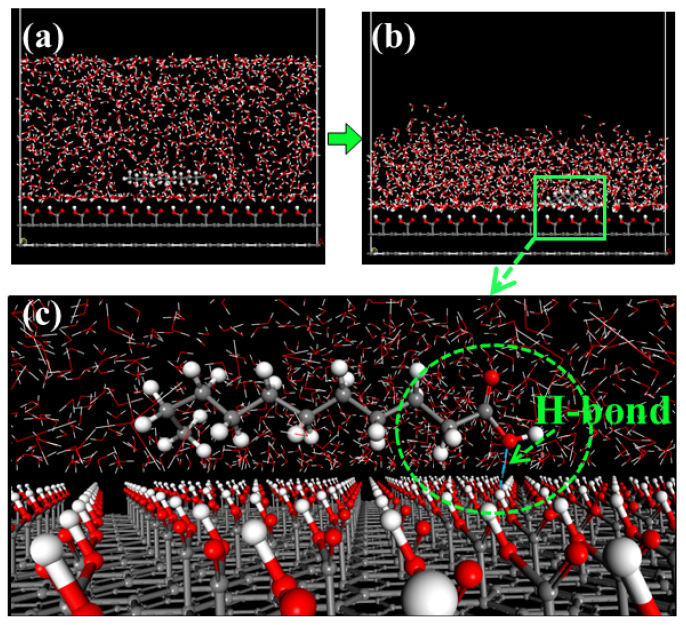
Adsorption of fatty acid collector molecules on a hydrophilic oxygenated carbonaceous surface: (**a**) initial structure; (**b**) balanced structure; (**c**) local enlargement.

**Figure 9:**
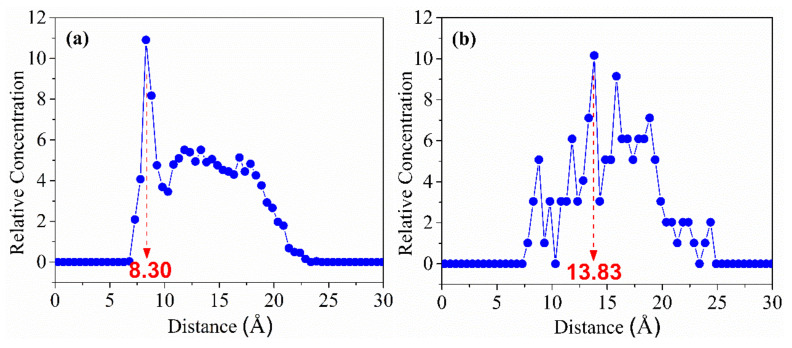
Water molecular concentration distribution at the interface: (**a**) nonpolar collector; (**b**) fatty acid collector.

The adsorption structure of nonpolar collector molecules on the hydrophilic oxygenated carbonaceous surface is shown in Figure 7. A large number of water molecules are adsorbed on the oxygenated carbonaceous surface through hydrogen bonds, and the surface water molecules have a higher density and more orderly arrangement than bulk water, which is known as a hydration film. The existence of this hydration film on the oxygenated carbonaceous surface makes the nonpolar molecules unable to be adsorbed on the surface. The adsorption structure of the fatty acid collector molecules on the oxygenated carbonaceous surface is shown in Figure 8. It was found that fatty acid molecules can adhere by forming hydrogen bonds between the head-group oxygen atoms and surface hydrogen atoms. This adhesion is relatively weak. To compare the hydrophobic modification effects of the two types of collectors, the water molecules near the collector molecules were selected as the target object, and the oxygenated carbonaceous surface was taken as the initial position. The relative concentration distribution of water molecules along the z-axis was calculated, as shown in Figure 9. It was observed that the adsorption morphology of water molecules and the oxygenated carbonaceous surface had an obvious peak at 8.30 Å in the nonpolar collector/oxygenated carbonaceous surface system, showing that the adsorption of water molecules was concentrated at this position, and the hydration film structure also appeared. In the fatty acid/oxygenated carbonaceous surface system, the adsorption morphology of water molecules and the oxygenated carbonaceous surface had a weak peak at 13.83 Å, and the water molecules were far away from the oxygenated carbonaceous surface, indicating that the fatty acid collector had the effect of hydrophobic modification on the oxygenated carbonaceous surface.

### 3.3. Analysis of AFM Force Measurement Results

**Figure 10:**
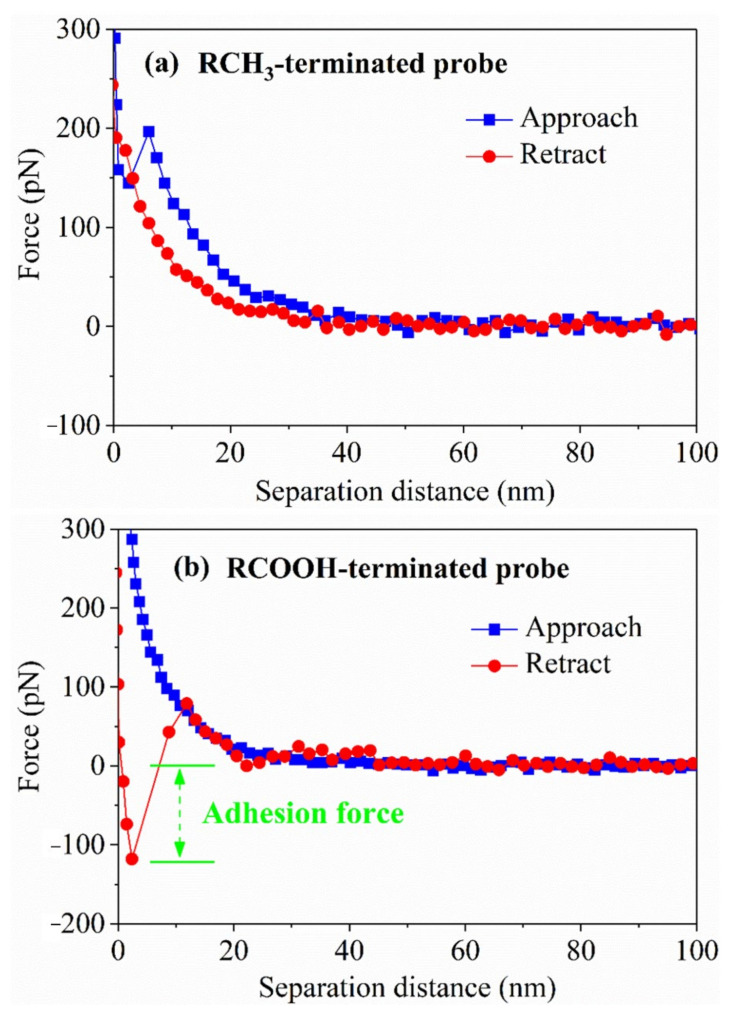
Interaction curves of RCH_3_-terminated and RCOOH-terminated probes with the hydrophilic surface.

The interaction curves between the RCH_3_-terminated and RCOOH-terminated probes and the hydrophilic surface were tested by AFM, as shown in Figure 10. The RCH_3_-terminated probe always showed repulsion in the approach process, which was mainly caused by electrostatic repulsion. There was no adhesion force in the retraction process, indicating that desorption was relatively easy, which verifies the result that the nonpolar collector cannot cover the hydrophilic point calculated by DFT. The RCOOH-terminated probe also always showed repulsion in the approach process. This is because the RCOOH-terminated probe had a long hydrophobic tail chain, which produced electrostatic repulsion when approaching the hydrophilic surface, and its repulsion was slightly lower than that of the RCH_3_-terminated probe. The RCOOH-terminated probe showed a noticeable adhesion force with a hydrophilic surface in the retraction process, indicating that fatty acids have a strong affinity for hydrophilic surfaces, and that fatty acids are more suitable as flotation collectors for low-rank coal than nonpolar hydrocarbon oil molecules. The DFT calculation results and AFM force test results confirmed one another, jointly revealing the interaction mechanism between fatty acids and oxygenated carbonaceous molecules, and providing a theoretical perspective for understanding the flotation performance of fatty acids at the micro level.

### 3.4. Changes in the Coal Surface Properties after Adsorption of Collectors

**Figure 11:**
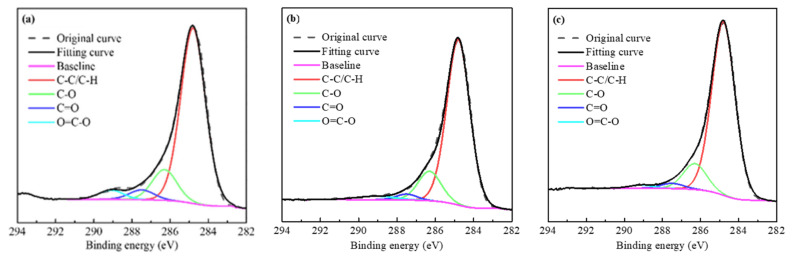
Peak fitting of C 1s peaks on the coal surfaces: (**a**) raw coal; (**b**) coal-octane; (**c**) coal-octanoic acid.

The C-C/C-H, C-O, C=O, and O-C=O peak fitting of C 1s peaks on the coal surfaces were conducted, as shown in Figure 11 and Table 2. It was observed that there were many oxygen-containing groups in the raw coal of low-rank coal, indicating that this kind of coal has strong hydrophilicity. After octane adsorption, the oxygen-containing groups on the coal surface decreased slightly. It should be noted that after adsorption of octanoic acid, the oxygen-containing groups on the coal surface decreased significantly. This shows that polar fatty acid collectors have an important impact on the properties of the coal surface, and verifies the conclusion that the RCOOH probe has a strong interaction with the coal surface in the AFM tests.

### 3.5. Implications for Low-Rank Coal Flotation

**Figure 12:**
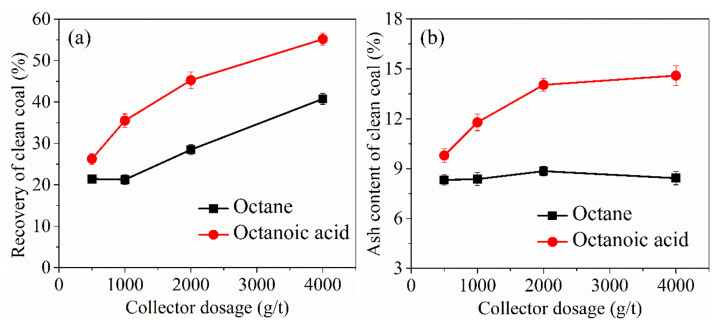
(**a**) Clean coal yield and (**b**) ash content of low-rank coal flotation using octane and octanoic acid as collectors.

Two hydrocarbon oil reagents-octane and octanoic acid-were used as collectors for the flotation separation of low-rank coal. The recovery of the flotation-separated clean coal and ash content is shown in Figure 12. The results show that the recovery of clean coal with fatty acids is significantly higher than that with a nonpolar collector. With an increase in the amount of collector, the recovery of clean coal increased. Although fatty acids can obtain a high clean coal yield, they also bring high clean coal ash and a poor separation effect, as shown in Figure 12b, as the fatty acid can easily interact with the surface of inorganic minerals, resulting in the floatation of inorganic minerals. Nonpolar hydrocarbon oil collectors are difficult to adsorb on the surface of low-rank coal, but fatty acids can adsorb well to achieve the surface modification of low-rank coal.

## 4. Conclusions

The following conclusions were drawn from this study: (1) The existence of a hydration film on the hydrophilic oxygenated carbonaceous surface prevents the adsorption of nonpolar molecules. The hydrophilic head groups of fatty acids have positive/negative electrostatic potential extreme points, which can be adsorbed on the negative electrostatic potential extreme points of the oxygenated carbonaceous surface through hydrogen bonds. The adsorption of fatty acids makes water molecules repel from the surface, indicating that the fatty acids have the effect of hydrophobic modification on the oxygenated carbonaceous surface.

Repulsion occurs when the RCH_3_-terminated probe approaches the hydrophilic surface, which is caused by electrostatic force, and there is no adhesion force in the retraction process. When the RCOOH-terminated probe approaches the hydrophilic surface, repulsion is caused mainly by electrostatic force, which is slightly lower than that of the RCH_3_-terminated probe. However, the RCOOH-terminated probe has a noticeable adhesion force with the hydrophilic surface in the retraction process, indicating that the fatty acid has a strong affinity for the hydrophilic surface. The flotation results show that fatty acids are more effective than nonpolar collectors. It should be noted that polar collectors also lead to high ash content of the concentrate. In the future, the combination of polar collectors and depressors can be considered to improve the yield and reduce the ash content of the concentrate.

## Figures and Tables

**Table 1:** Particle size distribution.

Size (mm)	Yield (%)	Ash (%)
0.5~0.25	39.93	14.16
0.25~0.125	17.12	11.76
0.125~0.074	13.74	12.80
0.074~0.045	9.09	12.93
<0.045	20.12	24.16
Total	100.00	15.46

**Table 2:** Peak split results of C 1s peaks of raw coal, coal-octane, and coal-octanoic acid.

Coal	Carbon Form (Relative % of C 1s)			
	C-C/C-H	C-O	C=O	O=C-O
Raw coal	77.89	13.66	4.36	4.10
Coal-octane	80.20	14.30	2.58	2.92
Coal-octanoic acid	83.42	12.48	2.50	1.60

## Sample Availability

Samples of the compounds are not available from the authors.
